# Compassion fatigue and compassion satisfaction among palliative care health providers: a scoping review

**DOI:** 10.1186/s12904-021-00784-5

**Published:** 2021-06-23

**Authors:** Manal Hassan Baqeas, Jenny Davis, Beverley Copnell

**Affiliations:** School of Nursing and Midwifery, La Trobe University, Bundoora, VIC 3086 Australia

**Keywords:** Compassion fatigue, Compassion satisfaction, Palliative care, Palliative care health providers, Scoping review

## Abstract

**Background:**

Palliative care can be demanding and stressful for providers. There is increasing recognition in the literature of the impact of caregiving in palliative care settings, including compassion fatigue and compassion satisfaction. However, to date this literature has not been systematically reviewed. The purpose of this scoping review was to map the literature on compassion fatigue and compassion satisfaction among palliative care health providers caring for adult patients.

**Methods:**

Scoping review method guided by Joanna Briggs Institute guidelines was conducted using four electronic databases to identify the relevant studies published with no time limit. Following the title and abstract review, two reviewers independently screened full-text articles, and extracted study data. A narrative approach to synthesizing the literature was used.

**Results:**

Twenty studies were included in the review. Five themes emerged from synthesis: conceptualisation of compassion fatigue and compassion satisfaction; measurement of compassion fatigue and compassion satisfaction; consequences of compassion fatigue or compassion satisfaction and providing care for patients with life-threatening conditions; predictors or associated factors of compassion fatigue and compassion satisfaction among palliative care health providers; and strategies or interventions to support palliative care health providers and reduce compassion fatigue.

**Conclusions:**

Limited studies examined the effectiveness of specific interventions to improve compassion satisfaction and reduce compassion fatigue among palliative care health providers. Further investigation of the impacts of compassion fatigue and compassion satisfaction on palliative care health providers and their work is also needed.

## Background

Palliative care aims to support people with life-threatening conditions and improve their quality of life [1]. Palliative care health providers (PCHP) comprise medical, nursing, and allied health care professionals who work in palliative care settings and who have specific knowledge, skills, and expertise in providing care for people living with a life limiting illness and their families. PCHP can provide direct care in various settings such as dedicated hospital wards, hospices, and community, and through consultancy to patients in other areas [2].

Prolonged contact with these patients predisposes PCHP to emotional and psychological distress such as compassion fatigue. There are various definitions of compassion fatigue documented in the literature. In general, compassion fatigue is a term used to describe the exhaustion that results from prolonged exposure to compassion stress among those who work in a caring profession [3]. Compassion fatigue is also described as the diminished ability to feel compassion or empathize when providing care. In contrast, compassion satisfaction is related to the pleasure derived from alleviation of patient suffering and positive work experience [4]. There is no consensus in the literature on the dimensions or components of compassion fatigue. However, there is a general agreement that compassion fatigue is related to both burnout (BO) and secondary traumatic stress (STS). While STS is very closely related to compassion fatigue, the nature of the relationship is defined differently and both terms used interchangeably by some authors [4]. The concept of compassion satisfaction is related to positive work experience, whereas compassion fatigue is associated with physical and emotional exhaustion, caused by constant, progressive, and cumulative negative experiences associated with various clinical settings [3, 5, 6]. Compassion fatigue has negative impacts on job satisfaction and patient outcomes [7–9]. This emphasizes the significance of investigating compassion fatigue in PCHP.

To date, compassion fatigue has been widely studied in health care providers in a range of settings, as synthesized in a recent meta-narrative review [10]. However, to our knowledge, no such synthesis has been undertaken of literature pertaining specifically to PCHP. This gap in the literature makes it difficult to identify and implement interventions to support these workers. Therefore, the aim of this scoping review is to synthesize findings from extant research about compassion fatigue and compassion satisfaction among PCHP.

## Methods

The scoping review, as a method, is suitable when the study topic is abstract, broad, emerging, or multi-dimensional [11]. Scoping reviews are used to answer a broad question such as “what is known about the study concepts?” [11]. It was, therefore, deemed suitable to address the aim of the current study. It answers the research question through a narrative synthesis of the literature. In addition, it is used to summarize the current knowledge about a topic and identify knowledge gaps regardless of the quality of reviewed studies and their design [11].

The current scoping review was conducted based on the guidelines published by the Joanna Briggs Institute (JBI) [12]. These guidelines were developed based on the previous work by Arksey and O’Malley [13] and Levac, Colquhoun, and O’Brien [14]. In addition, the literature review followed the PRISMA-ScR checklist to provide clear details of the search protocol and enhance methodological transparency [11]. As per the Joanna Briggs Institute guidelines, the following five stages were followed: 1. Identifying the research question 2. Identifying relevant studies 3. Selection of relevant studies 4. Charting the data 5. Collating, summarizing and reporting the results [12]. There is a sixth (optional) step that includes consultation with key stakeholders. This step was omitted, however, and only evidence published in peer-reviewed literature was included.

### Stage 1. Identifying the research question

This review aims to identify what is known about compassion fatigue and compassion satisfaction among PCHP. To address the study aim, the review was conducted to answer the following question: “what research has been undertaken on compassion fatigue and compassion satisfaction among palliative care health providers?”

### Stage 2. Identifying relevant publications

The review was conducted by a team of researchers including the primary researcher, content experts, and methodological experts. A search of four electronic databases: MEDLINE (OVID), CINAHL, PsycInfo, and EMBASE was conducted in August 2019. To ensure a comprehensive search, the search terms “compassion fatigue”, “compassion satisfaction”, and “palliative care health providers” were initially kept broad and then exploded to cover MeSH terms. In addition, keywords included in the title and abstract of retrieved papers, and the keywords used to describe the articles were identified. These keywords were searched across the databases. Finally, the reference lists of the selected articles were hand searched to identify additional studies. The terms “compassion fatigue”, “compassion satisfaction”, and “palliative care health providers” were combined with the following terms: “burnout, professional”, “stress disorders, post-traumatic”, “fatigue, compassion”, “secondary trauma”, “secondary traumatic stress”, “secondary traumatization”, “trauma, vicarious”, “traumas, secondary”, “traumatic stress, secondary”, “burnout, career’, “burnout, occupational”, “burnout, professional”, “secondary post-traumatic stress”, “hospice professionals”, “hospice, palliative care nursing”, “palliative care”, “palliative medicine”, “terminal care”, “palliative supportive care”, and “palliative treatment”. The Boolean operators ‘AND’ and ‘OR’ were used to combine various terms and concepts. All identified sources were stored in the EndNote reference program. Irrelevant records and duplicates were excluded from the literature search. The final screening of title/abstract and then full text was managed in Covidence.

Inclusion criteria were: 1. all research designs (e.g., quantitative, qualitative, mixed methods, and systematic reviews); 2. addressing compassion fatigue and compassion satisfaction from the perspectives of PCHP caring for adult patients in any practice setting; 3. published in English with no date limits applied. Exclusion criteria were: 1. grey literature (e.g., book chapters, theses, reports, and conference abstracts); 2. Non-research publications (eg editorials; discussion papers; opinion pieces); 3. targeting volunteers working in palliative care settings; 4. investigating BO without STS or Compassion Fatigue; 5. focusing on PCHP working with pediatric patients as we consider pediatric palliative care has distinct differences from adult palliative care and can be considered a speciality in its own right [15].

### Stage 3. Publication selection

After removal of duplicates, article titles and abstracts were screened by two researchers independently. Disagreements were discussed and resolved by consensus among the research team. After full text screening, studies meeting all inclusion criteria were included in the final review.

### Stage 4. Charting the data

A data extraction table was used to extract the data from the included studies. Extracted data included country, year of publication, names of authors, study purpose, research design, study sample, and main study findings. The data extraction was conducted by one researcher and reviewed by the research team. Any disagreements in data extraction were resolved by consensus. References were managed utilising EndNote (version X9) and included studies were imported to Covidence during the final screening. In line with the PRISMA-ScR standards [11], no formal quality appraisal was undertaken as it was not intended to exclude any paper based on quality assessment.

### Stage 5 data synthesis

Narrative synthesis was employed due to the heterogeneity of the studies. The characteristics of the reviewed studies (i.e design, sample, settings, main variables, and publication year) were collated and summarized. Studies were summarized in a Table and a content analysis was performed based on the tabulated data. Then, contents were translated into main themes. Lastly, the findings were interpreted and compared with studies from other settings.

## Results

**Fig. 1:**
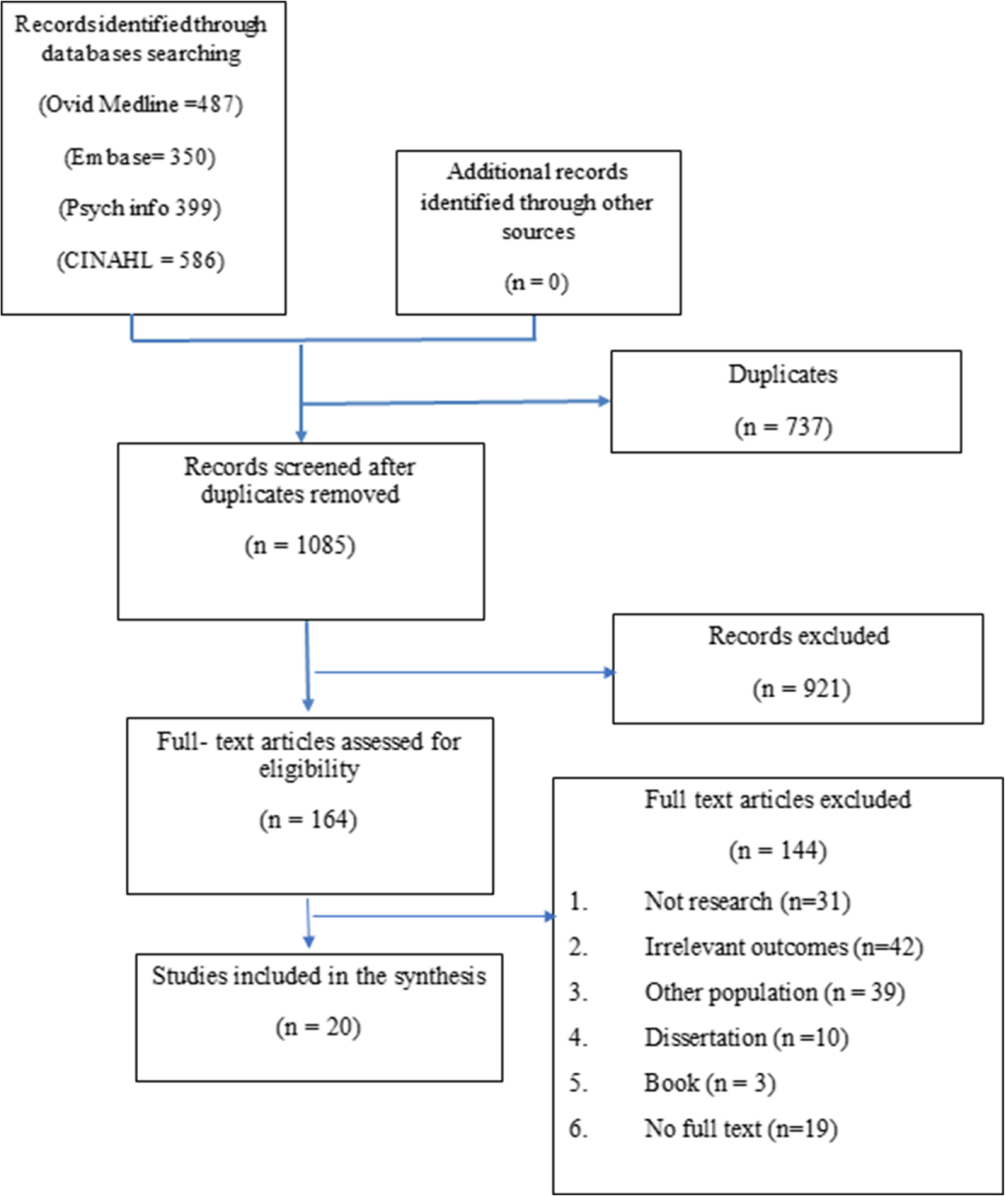
Flow diagram of search strategy

Overall, the initial search yielded 1822 records. After removing duplicates, 1085 records were screened for potential relevance by title and abstract. Of these, 921 records were found to be irrelevant and 164 full-text articles were screened. Finally, 144 articles were excluded and 20 articles were included in the final review (Fig. 1). Studies were conducted in different countries worldwide, the majority in a Western setting. Countries represented were: United States (*n* = 9), Spain (*n* = 3), Israel (*n* = 3), with one study from each of Australia, Canada, New Zealand, and India. The majority of the studies were published within the last 5 years (*n* = 15). More than half of the studies were correlational (*n* = 10), four studies were qualitative, one a quantitative descriptive study, one a pre-post study with control group, one pre-post with no control group, two studies examined the psychometric properties of the Professional Quality of Life (ProQOL) scale, one paper was a systematic review. Study populations included PCHP from several disciplines (*n* = 14), only nurses (*n* = 3), or only physicians (*n* = 2).

The samples in the included studies were recruited from various settings that provide palliative care (Table 1). One study was conducted in inpatient hospices and hospitals [20]. One study was conducted in inpatient hospices [21], one in outpatient hospices [1], and one in hospice settings without specifying whether inpatient or outpatient [22]. One study was conducted in outpatient palliative care setting [24]. Eight studies included participants from both inpatient and outpatient settings including hospices [16, 18, 19, 23, 28–30, 33]. However, the combination between inpatient and outpatient settings in these eight studies was unclear and not described in detail. Six studies included participants from settings that provide inpatient and outpatient services without stating specifically if all participants were recruited from inpatient, outpatient, or both [17, 25–27, 31, 32].

**Table 1:** Summary Table of Included Studies

Authors, Year, and Country	Settings	Design	Sample	Research Aims	Outcomes
Alkema et al. [1], USA	Outpatient palliative care settings: home hospice settings.	Quantitative (Cross sectional survey)	*n* = 37 Hospice Professionals including 17 Registered Nurse 5 Home Health Aide 4 Social Worker 2 Volunteer Coordinator 3 Bereavement Professional 2 Chaplain 1 Administrative Assistant 2 Medical Director 2 Other.	Examine the relationships among self-care, compassion fatigue, compassion satisfaction, and BO among hospice care workers.	Self-care strategies were associated with decreased levels of compassion fatigue and BO and higher levels of compassion satisfaction.
Barnett, Ruiz [16], USA	Both inpatient and outpatient palliative care settings Inpatient – hospices, hospitals, nursing homes, other long-term care facilities, Outpatient – home healthcare.	Quantitative (Cross sectional survey)	90 hospice nurses.	To study the role of self-esteem in mediating the relationship between compassion fatigue and psychological distress among hospice nurses.	Psychological distress can decrease self-esteem, and thereby increase the risk of compassion fatigue.
Bessen et al. [17], USA	Medical centre – unable to determine if includes both inpatient and outpatient settings.	Qualitative (semi-structured interviews)	13 physicians.	To describe compassionate care provision by physicians during end-of-life care.	There were variable ways for delivering compassionate care. Physicians need training in end-of-life care to overcome some barriers of providing care on organizational and individual level.
Frey et al. [18], New Zealand	Inpatient – hospital, hospice, residential aged care, Outpatient – hospital, community hospice, district nursing, general practice, other community services.	Quantitative (Cross sectional survey)	256 registered nurses.	To investigate BO and compassion fatigue and their associated factors among nurses in New Zealand.	BO negatively associated with psychological empowerment & commitment & challenge components of psychological hardiness. STS negatively associated with palliative care education. Compassion satisfaction positively associated with palliative care education, psychological empowerment, & both commitment & challenge components of psychological hardiness.
Galiana et al. [19], Brazil and Spain.	Inpatient and outpatient palliative care settings including hospice (Home-based palliative care Social-health center unit palliative care Hospital support team Hospice Oncology unit Intensive treatment unit Pediatrics unit of palliative care Others)	Psychometrics	Brazil/ (*n* = 161) Spain/ (*n* = 385) PCHP including doctors, nurses, psychologists, nursing assistants, social workers and other. (Individual providers number not specified)	To assess the reliability and validity of the Spanish and the Portuguese versions of the ProQOL scale.	Both Spanish and Portuguese versions of the ProQOL show good psychometric properties.
Heeter et al. [20], USA	Inpatient hospice and hospital palliative care settings	(Pre-Post) one group	36 Hospice and PCHP including nurses, managers from the respective home hospice and palliative care units, physicians, clerical, aides, social workers, and others. (Individual providers number not specified)	Examine the effects of 6-week technology-assisted meditation program on emotional awareness, compassion fatigue, and BO	The 6-week technology-assisted meditation technology successfully reduced compassion fatigue/BO and increased emotional awareness among the study participants.
Hill et al. [10]	Various settings	Systematic Review	547 PCHP across 9 studies	To explore the effectiveness of interventions used to enhance psychological wellbeing of palliative care staff.	Few interventions were helpful to support palliative care staff and improve their well-being.
Hilliard [21], USA	Inpatient palliative care settings: hospice	Two groups pre-post-test group	*n* = 17 nurses, social workers, and chaplains (Individual providers number not specified)	To examine the effectiveness of music therapy to reduce compassion fatigue and improve team building of hospice workers.	Music therapy was effective to improve team building but not reduce compassion fatigue.
Hotchkiss [22], USA	Hospice settings from VITAS® Healthcare	Quantitative (Cross sectional survey)	324 Hospice care professionals including 68 Registered nurse 60 Chaplain 48 Social worker 40 Home health aid 28 Licensed vocational nurse 20 Administrative 16 Management 14 Nurse practitioners 8 Physician 4 Music therapists 18Other	Examine the relationship between compassion satisfaction, BO, STS, and mindful self-care	Participants had high levels of self-care and compassion satisfaction and low levels of STS and BO. Self-care strategies can improve compassion satisfaction.
Kaur et al. [23], India	Inpatient and Outpatient palliative care settings including hospice (hospital and hospice at cancer palliative care centers)	Quantitative (Cross sectional survey)	65 PCHP including doctor, nurse, counselor, psychologist, social worker, pharmacist, or physiotherapist. (Individual number not specified).	To explore the professional quality of life among PCHP.	The authors concluded that implementing specific interventions could be helpful to reduce STS and BO and enhance compassion satisfaction among PCHP.
Melvin [24], USA	Outpatient palliative care settings: home health agency	Qualitative (semi-structured interviews)	6 palliative care nurses	Assess prevalence of compassion fatigue, its consequences, and methods of coping with it among palliative care nurses.	Compassion fatigue had negative physical and emotional health impacts on palliative care nurses.
Montross-Thomas et al. [25], USA	Can not determine the participants were recruited online through a membership list serve of the National Hospice and Palliative Care Organization (NHPCO). All participants were hospice staff or volunteers who were emailed a description of the study and a Survey Monkey link	Quantitative (Cross sectional survey)	390 hospice staff and volunteers (Individual providers not specified)	To investigate the role of practicing rituals to improve professional quality of life among hospice care providers	Hospice care providers who practice rituals were found to have better professional quality of life.
Mota Vargas et al. [26], Spain	Can not determine (purposeful sample)	Qualitative (semi-structured individual interviews)	10 PCHP include nurses, doctors and psychologists. Individual providers number not specified).	To describe experiences of palliative care workers over time	PCHP were found to go through various phases during their professional life.
O’Mahony et al. [27], USA	Can not determine Participants were recruited from a group of 70 professionals participating in a continuing education program on palliative medicine in Midwest Academic Medical Center	Quantitative (Cross sectional survey)	66 PCHP including physicians, nurses, chaplains, social workers, and other. (Individual providers number not specified)	Examine the relationship between personality traits and compassion fatigue in PCHP.	Neuroticism was associated with STS and BO. Agreeableness was associated with compassion satisfaction. Experience in palliative care was associated with lower levels of BO and increased levels of compassion satisfaction.
Samson, Shvartzman [28], Israel	Both inpatient and outpatient palliative care settings (hospital-based and/or home-based palliative care units)	Quantitative (Cross sectional survey)	144 PCHP including 47 Physicians 97 Nurses	To identify the association between STS and peritraumatic dissociation among palliative workers.	STS was found to be significantly correlated with clinical levels of peritraumatic dissociation among palliative workers.
Samson, Shvartzman [29], Israel	Both inpatient and outpatient palliative care settings (end-of-life community- and hospital-based palliative care units)	Quantitative (Cross sectional survey)	241 participants providing palliative care and primary care including 84 Physician 157 Nurse	To assess the relationship between exposure to death and dying and professional quality of life in PCHP	There was a significant relationship between exposure to death and dying and professional quality of life among the study participants.
Samson et al. [30], Israel	Both inpatient and outpatient palliative care settings including hospice (home- and hospital-based hospice units and of primary health care providers, working in Clalit Health Care Services and Maccabi Health Care Services (the two largest health care organizations in Israel)	Quantitative (psychometric properties)	1100 health care providers	To assess the reliability and validity of the Hebrew version of the 30-item ProQol scale	The Hebrew version of the compassion satisfaction subscale was found to show good psychometric properties
Sansó et al. [31], Spain	Can not determine settings: member e-mail list of the Spanish Society of Palliative Care	Quantitative (Cross sectional survey)	387 PCHP include doctors, nurses, psychologists, nursing assistants, social workers. (Individual providers number not specified)	To assess the relationships among Self-Care, Awareness, professional quality of life, and Coping with Death among PCHP.	There was a significant relationship among the study variables consistent with the awareness-based model of self-care.
Slocum-Gori et al. [32], Canada	Can not determine: The Canadian Hospice Palliative Care Association (CHPCA) provided their membership mail-out for contacting managers and administrators of HPC organizations.	Quantitative (Cross sectional survey)	630 PCHP including clinical, administrative, allied health workers and volunteers. (Individual providers number not specified)	Examine the relationships among compassion fatigue, compassion satisfaction, and BO among palliative care workers	Compassion satisfaction was negatively associated with BO and compassion fatigue. BO and compassion fatigue were positively associated. Compassion satisfaction, BO, and compassion fatigue differed significantly according to some characteristics of the sample.
Zambrano et al. [33], Australia.	Inpatient and outpatient palliative care settings including hospice (inpatient unit/ hospice, a consultation liaison into tertiary and other hospitals in the region and a community outreach program)	Qualitative (one-on-one interview)	7 palliative medicine physician specialists	To assess experiences of palliative medicine specialists who provide care for dying patients, as well as the impact of providing care for these patients	The study participants were at high risk for compassion fatigue. However, they used some coping strategies that reduced their stress.

### Themes extracted from the included studies

Five main themes were identified in the synthesis of the included studies: 1. conceptualisation of compassion fatigue and compassion satisfaction; 2. measurement of compassion fatigue and satisfaction; 3. consequences of compassion fatigue or satisfaction and of providing care for patients with life-threatening conditions; 4. predictors or associated factors of compassion fatigue and satisfaction among PCHP; 5. strategies or interventions to support PCHP and reduce compassion fatigue. These themes are described further in the following sections. The summary of the included studies is shown in Table 1.

### Theme 1- conceptualisation of compassion fatigue

Overall, the reviewed studies did not discuss the conceptualisation of compassion fatigue in depth. Their definition was mainly embedded in that used by the measurement tool and thus reflects changes in the concept over time. Compassion satisfaction was defined by some studies as a positive consequence of providing care for acutely ill or traumatised patients (e.g., a sense of accomplishment and reward) [1, 18, 19, 22, 25, 30, 31]. Some studies treated compassion fatigue as a single discrete entity with no constitutive components [1, 21]. On the other hand, some studies treated compassion fatigue as being synonymous with STS, and these terms were used interchangeably [20, 31, 32]. The remaining studies conceptualized compassion fatigue as having two discrete components (STS and BO), each of which was measured separately [16, 18, 19, 22, 23, 25, 27–30]. The qualitative studies did not specify a clear definition of compassion fatigue [17, 24, 26, 33].

### Theme 2: measurement of compassion fatigue and satisfaction

The measurement tools used to assess compassion fatigue and compassion satisfaction among PCHP included the 30-item professional quality of life scale (ProQOL) scale, the 20-item compassion fatigue scale (CFS), and the 13-item Compassion Fatigue Short-Scale. The various versions of the ProQOL reflect the changes in conceptualisation described in the previous section. The ProQOL-V includes two domains of compassion fatigue (composed of BO and STS) and compassion satisfaction. The ProQOL-IV measures three domains: compassion satisfaction, BO, and compassion fatigue/secondary trauma. The ProQOL-III measures three domains: compassion satisfaction, BO, and compassion fatigue. The 20-item compassion fatigue scale (CFS) is a subscale of the 66-item Compassion Satisfaction/Fatigue Self-Test for Helpers which measures compassion satisfaction, compassion fatigue, and BO. The 13-item Compassion Fatigue Short-Scale measures compassion fatigue in two dimensions (secondary trauma and job BO).

The most commonly used measure of compassion fatigue and compassion satisfaction among PCHP was the ProQOL scale (III, IV, and V versions), which was used in 11 studies [1, 18, 20, 22, 23, 25, 27–29, 31, 32]. This scale measures compassion satisfaction, STS, and BO. The items of each subscale are rated on a five-point Likert-type scale. The scale has demonstrated excellent psychometric properties with Cronbach’s alpha of 0.80 or more for its subscales [4].

The Compassion Fatigue Scale (CFS) was used in only one study [21] which was a pre-post study. This tool is distinguished from the other tools by focusing more on the helper and working environment. In addition, the Compassion Fatigue Short-Scale was used in one study [16]. This tool measures only compassion fatigue. Both the 20-item CFS and the 13-item CFS were reported to have adequate reliability and validity [16, 21]. Therefore, all of the three tools have been utilised internationally with various populations. Apart from the psychometric properties of these three measurement tools, authors did not report any other evidence about their efficacy. In addition, they did not provide a rationale for their choice of these tools in their studies.

Four studies reported the levels of compassion fatigue and compassion satisfaction among PCHP. All four used the professional quality of life scale (ProQOL) scale. In the study of Frey et al., [18] about half (48.4%) of palliative care nurses had moderate to high levels of compassion satisfaction. However, about a quarter of the participants had high BO scores (26.8%) and more than half (51.6%) had moderate STS [18]. O’Mahony et al. [27] found that palliative medicine physicians had overall high levels of compassion satisfaction and low levels of BO and STS. Alkema, Linton, and Davies [1] found that the mean scores of compassion satisfaction, BO, and compassion fatigue among hospice professionals were in the average range. Finally, Kaur, Sharma, and Chaturvedi reported that, among palliative care providers, 49.2% had an average level of compassion satisfaction, 53.8% had an average level of BO, while 95.4% scored above 75th percentile on STS [23].

### Theme 3: consequences of compassion fatigue

Two studies, both qualitative, reported consequences of compassion fatigue among their findings. A study conducted by Melvin reported that providing palliative care and working with dying patients could contribute to compassion fatigue among PCHP [24]. The author also suggests that providing palliative care and working with dying patients could contribute to physical and emotional consequences. PCHP reported feeling responsible for patient care even after going home and leaving the workplace [24]. In addition to compassion fatigue, working with dying patients likely affects many dimensions concerning mental health including feelings of guilt, sadness, crying, thinking of death, remembering personal experiences with death, isolation, and grief [33].

### Theme 4: predictors or associated factors of compassion fatigue and satisfaction

Eleven articles provided data about the correlates of high levels of compassion fatigue and poor compassion satisfaction among PCHP. In general, studies included PCHP from several disciplines. However, two studies had only nurse samples and one study had both physician and nurse samples. The synthesis of these studies is included below.

In general, demographic, personal, and organisational factors were associated with compassion fatigue and compassion satisfaction among PCHP. Demographic factors were found to be associated with compassion fatigue in some studies. Slocum-Gori et al. [32] found that employment status was associated with compassion fatigue as part-time workers had lower scores than those who worked full time. Additionally, they found that greater experience in palliative care was associated with lower levels of BO. O’Mahony et al. [27] supported these results and found that duration of experience in palliative care was associated with higher levels of compassion satisfaction.

Personal factors were found to be associated with compassion fatigue in several studies. For example, having a neuroticism personality trait was associated with increased levels of STS and BO among PCHP, while having an agreeableness personality trait was associated with increased levels of compassion satisfaction [27]. In addition, psychological hardiness (e.g., commitment and challenge) were associated with lower BO and greater compassion satisfaction [18]. Furthermore, practicing some personal rituals on specific occasions was associated with lower BO and more compassion satisfaction among hospice staff [25]. Also, the ability to cope with death was associated with lower levels of compassion fatigue and BO and higher compassion satisfaction among PCHP [31]. Greater exposure to death was also significantly correlated with STS among physicians and nurses employed in a palliative care unit [29]. In addition, high levels of dissociation (detachment) were associated with higher levels of STS [28]. Psychological distress was also associated with increased compassion fatigue [16]. Further, using self-care strategies was associated with lower levels of compassion fatigue and BO and higher levels of compassion satisfaction [1]. Further, mindful self-care was associated with more compassion satisfaction and less risk of BO among health care workers in the palliative care setting [22].

Frey et al. found that organizational factors such as work-related empowerment could decrease BO levels [18]. Furthermore, the authors found that STS was negatively associated with previous palliative care education [18]. Kaur et al. concluded that receiving training in palliative care was associated with lower levels of BO and STS [23]. Kaur et al. found that professional orientation was associated with compassion satisfaction, with nurses scoring lower levels than other health professionals [23]. Slocum-Gori et al. [32] found that compassion fatigue was negatively correlated with compassion satisfaction and positively correlated with BO.

### Theme 5: strategies or interventions to support PCHP or reduce compassion fatigue

In one systematic review, Hill et al. identified multiple interventions reported to improve wellbeing of PCHP; however, most were found to be ineffective in reducing compassion fatigue [34]. Examples of these interventions include cognitive training, education, relaxation, and support [34]. Two of the included studies evaluated interventions to reduce compassion fatigue among PCHP. The first study by Heeter, Lehto, Allbritton, Day and Wiseman examined the effectiveness of a 6-week meditation program delivered via smartphone apps to reduce compassion fatigue among 36 PCHP [20]. The single group pre and post-test study design reported a significant reduction in compassion fatigue after the intervention [20]. Another study conducted by Hilliard [21] investigated the effectiveness of a music therapy intervention to reduce compassion fatigue in a sample of 17 hospice workers. Participants were randomly assigned to an ecological music therapy group and a didactic music therapy group. A pre-and post-test was performed to measure compassion fatigue levels. The results indicated no significant differences in compassion fatigue between pre-and post-test scores of compassion fatigue in either group [21].

Four qualitative studies reported strategies to support PCHP from the perspectives of the study participants [17, 24, 26, 33]. These studies did not actually measure the effectiveness of these strategies. However, the researchers interviewed PCHP and asked them to list strategies they believed helped to protect them from compassion fatigue. Palliative care nurses in the study by Melvin described adopting various strategies including setting professional boundaries, seeking support from colleagues and supervisors, reflection, physical exercise, and social activities out of work [24]. In the study of Mota Vargas et al. researchers interviewed PCHP and asked them to identify the self-care strategies they used [26]. Participants reported that reflecting on their experience of providing palliative care, understanding the methods used to enhance self-control, and acknowledging one’s limits and accepting the fact that many things cannot be changed and learning to live with them were the most commonly used strategies. Other self-care strategies included attending training in palliative care, improving their communication skills, and developing personal hobbies [26]. Zambrano, Chur-Hansen, and Crawford reported that PCHP highlighted supportive measures such as finding spiritual meaning, receiving support, and using both problem-focused and emotion-focused coping strategies [33]. Bessen, Jain, Brooks et al. reported that physicians described sharing experiences with their colleagues or using individual-based strategies (e.g., improving self-awareness) to prevent compassion fatigue [17].

## Discussion

This scoping review mapped available evidence on compassion fatigue and compassion satisfaction among PCHP in various palliative care settings. The current scoping review included all relevant studies regardless of the publication year but the majority that met inclusion criteria were published within the last 5 years (*n* = 16). This suggests that interest in compassion fatigue and compassion satisfaction in the field of palliative care is increasing.

Themes that emerged in this review were also reported by previous reviews focusing on other health professionals in non-palliative care settings. In a meta-narrative review related to compassion fatigue in health literature, the main themes that emerged were related to predictors/risk factors of compassion fatigue, its consequences, conceptualization, and measurement [10]. Another review related to compassion fatigue in cancer care providers included themes related to compassion fatigue prevalence, measurement, and management [35]. These reviews reported various predictors/risk factors and consequences of compassion fatigue that are, to some extent, similar to these reported in the current study.

Findings in our review suggest a general agreement that compassion satisfaction reflects a sense of accomplishment and reward of providing care for patients [1, 18, 19, 22, 25, 30, 31]. However, there was no consensus on the definition of compassion fatigue in palliative care settings. While some studies treated compassion fatigue as a single discrete entity, or synonymous with STS [20, 31, 32], it was considered a multi-dimensional concept by others [16, 18, 19, 22, 23, 25, 27–30]. The multi-dimensionality of compassion fatigue is further complicated because it is informed by different theories that inform the definition of compassion fatigue [10, 36]. This renders the development of a unified meaning of compassion fatigue difficult. This also resulted in the variability of the domains or subscales of the measures used to assess compassion fatigue. Most of the included studies used the ProQOL scale which assessed BO and STS as components of compassion fatigue rather than reporting an overall score for compassion fatigue.

Compassion is a central concept for PCHP who provide care for people with life limiting conditions. The more empathic a palliative care provider becomes, the more likely compassion fatigue will occur. Therefore, it is important to educate PCHP to modify empathetic ability in response to prolonged work with patients needing palliative care. The human nervous system plays an important role in regulating the empathetic response of the individual. Recent literature has shown that empathy is influenced by nervous system stimulation and it may lead to empathic distress [37].

The literature review revealed various organizational factors (e.g., work-related empowerment, receiving training in palliative care, and being recognized as a palliative care nurse) and demographic factors (e.g., employment status as part-time workers or full time and experience in palliative care) associated with compassion fatigue and compassion satisfaction across PCHP. Further, it was noted that some personal factors associated with compassion fatigue and compassion satisfaction were nonmodifiable (e.g., neuroticism personality trait and psychological hardiness). Additional factors included personal variables such as practicing some personal rituals, the ability to cope with death and self-care, levels of dissociation, using self-care strategies and mindful self-care. Therefore, it can be concluded that compassion fatigue and compassion satisfaction are predicted by many factors, some of which may not be modifiable.

The majority of studies included participants from multiple work settings (hospital, hospice and community) and none compared findings across settings or attempted to differentiate between them. Given that work in the various settings can vary considerably, the incidence and experience of compassion fatigue may also vary. Future research should explore the impact of work setting on compassion fatigue and compassion satisfaction.

Receiving palliative care training or education was found to help reduce the likelihood of developing symptoms of compassion fatigue [18, 23, 38]. None of these studies explored the content of education programs to identify which aspects induced this effect. Studies in non-palliative care settings have investigated training programs specifically focused on reducing or preventing compassion fatigue. For example, in a Pre- Post- test study conducted to examine the effect of Mindful Self-Compassion (MSC) training on compassion fatigue and resilience among nurses working in various settings, there was a significant reduction in the scores of secondary trauma and BO after the intervention [39]. Another study reported a significant reduction in participants’ compassion fatigue and BO and improvement in compassion satisfaction after Compassion Fatigue Specialist Training for mental health professionals [40]. It would seem likely given the nature of palliative care work that specialist education programs would include a focus on similar self-care activities; an examination of the curricula of these programs would be useful in explicating this content. We recommend that PCHP undergo specific education/training in this area, whether through formal programs or continuing professional development.

A number of interventions have been shown to reduce compassion fatigue and improve compassion satisfaction across a wide range of populations [39, 40]. However, few intervention studies were conducted specific to the field of palliative care. Only two of the included studies in this review involved interventions and measured their effectiveness to mitigate compassion fatigue and improve compassion satisfaction among PCHP. Only one of the tested interventions (The 6-week technology-assisted meditation) was found to be effective in reducing compassion fatigue. Despite this, many descriptive or correlational studies pointed to such interventions. Other studies investigated strategies to support PCHP using self-report data with correlational or qualitative approaches rather than actually implementing these strategies or measuring their effectiveness [17, 24, 26, 33]. Therefore, most of the knowledge regarding the interventions used to mitigate compassion fatigue and improve compassion satisfaction among PCHP is informed by low level evidence. Furthermore, while there is some overlap between palliative care and other health care specialties, there are also aspects that are unique to palliative care. Therefore, it cannot be assumed that research undertaken in other specialty areas can be applied to PCHP, and we recommend interventions be tested in this population.

## Strengths and limitations

The strengths of this review include conducting a comprehensive search with no limits on publication dates. In addition, studies that used concepts related to compassion fatigue but did not examine the concept directly (e.g., empathy, moral distress) were excluded from the literature search to make the search methodology more rigorous. Nevertheless, the review has some limitations. First, some relevant studies may have been missed despite using a rigorous search strategy. This could occur due to the complexity of compassion fatigue terms and inconsistencies in its conceptualisation across different studies. Second, only publications written in English were included which limits generalisability and may introduce language bias. The limited number of studies examining compassion fatigue in palliative care settings may warrant conducting a broad search in all languages. Grey literature was excluded, which may introduce publication bias.

The results of this review highlight a gap in the literature examining impacts of compassion fatigue and compassion satisfaction on PCHP. This gap in the literature demonstrates the need for further research on the impacts of compassion fatigue and compassion satisfaction on PCHP. Therefore, as nurses make up a significant proportion of the palliative care health provider workforce, we recommend exploring the impact of compassion fatigue and compassion satisfaction on productivity among palliative care nurses. Targeting a homogeneous sample of nurses is also recommended since the included studies predominantly involved heterogenous samples of PCHP rather than specifically nurses. Research is also required to understand whether and how the experience of compassion fatigue and compassion satisfaction may vary across different work environments. In addition, there is a need to conduct interventional studies to identify the most effective strategies, including education or training, to reduce compassion fatigue among PCHP.

## Conclusion

This review sought to identify current evidence about compassion fatigue and compassion satisfaction among PCHP. Most of the studies investigating the impacts of compassion fatigue and compassion satisfaction on PCHP were descriptive in nature. This indicates a gap in the literature that needs more investigation. Only one study identified an effective intervention to reduce compassion fatigue in PCHP. Most of the reviewed studies were correlational or exploratory in nature which affects the quality and strength of the retrieved evidence. One important aspect to be considered is the impact of compassion fatigue and compassion satisfaction on the productivity of PCHP and their ability to provide safe and compassionate care. This is an important topic especially among palliative care nurses since they are the largest group of PCHP and they spend a long time caring for people with life-threatening conditions and related trauma. The current work suggests a need to fill various gaps in knowledge and provides a clear direction for future research.

## Data Availability

Data used in this manuscript consist of published articles which cannot be shared by the authors for copyright reasons but are available through subscription to the relevant journals/databases.

## Abbreviations

PCHP: Palliative care health providers
BO: Burnout
STS: Secondary traumatic stress
ProQOL: Professional quality of life
CFS: Compassion fatigue scale
